# Individualized positive end-expiratory pressure guided by end-expiratory lung volume in early acute respiratory distress syndrome: study protocol for the multicenter, randomized IPERPEEP trial

**DOI:** 10.1186/s13063-021-05993-0

**Published:** 2022-01-20

**Authors:** Domenico Luca Grieco, Salvatore Maurizio Maggiore, Giacomo Bellani, Savino Spadaro, Elena Spinelli, Tommaso Tonetti, Luca S. Menga, Marco Pozzi, Denise Battaglini, Rosa Di Mussi, Andrea Bruni, Andrea De Gaetano, Carmine Giovanni Iovino, Matteo Brioni, Francesco Mojoli, Giuseppe Foti, Carlo Aberto Volta, Paolo Pelosi, Paolo Navalesi, Salvatore Grasso, V. Marco Ranieri, Massimo Antonelli, the IPERPEEP study group

**Affiliations:** 1 Department of Emergency, Intensive Care Medicine and Anesthesia, Fondazione Policlinico Universitario A. Gemelli IRCCS, L.go F. Vito, 00168 Rome, Italy; 2 Istituto di Anestesiologia e Rianimazione, Università Cattolica del Sacro Cuore, L.go F. Vito, 00168 Rome, Italy; 3 Department of Medical, Oral and Biotechnological Sciences, School of Medicine and Health Sciences, Section of Anesthesia, Analgesia, Perioperative and Intensive Care, SS. Annunziata Hospital, Gabriele d’Annunzio University of Chieti-Pescara, Chieti, Italy; 4 Department of Medicine and Surgery, University of Milan-Bicocca, Via Cadore 48, Monza, Italy; 5 Department of Emergency and Intensive Care, San Gerardo Hospital, Monza, Italy; 6 Department Morphology, Surgery and Experimental medicine, Anesthesia and Intensive care section, University of Ferrara, Azienda Ospedaliera-Universitaria Sant’Anna, Ferrara, Italy; 7 Dipartimento di Anestesia, Rianimazione ed Emergenza-Urgenza, Fondazione IRCCS Ca’ Granda Ospedale Maggiore Policlinico, Università degli studi di Milano, Milan, Italy; 8 Dipartimento di Scienze Mediche e Chirurgiche, Anesthesia and Critical Care Medicine, Policlinico di Sant’Orsola, Alma Mater Studiorum University of Bologna, Bologna, Italy; 9 Anesthesia and Intensive Care, Fondazione IRCCS Policlinico San Matteo, Università degli Studi di Pavia, Pavia, Italy; 10 Department of Surgical Sciences and Integrated Diagnostics, DISC, University of Genoa, Genoa, Italy; 11 Anesthesia and Intensive Care, San Martino Policlinico Hospital, IRCCS for Oncology and Neurosciences, Genoa, Italy; 12 Department of Emergency and Organ Transplant (D.E.T.O.), University of Bari Aldo Moro, Bari, Italy; 13 Anesthesia and Intensive Care Unit, Department of Medical and Surgical Sciences, University Hospital Mater Domini, Magna Graecia University, Catanzaro, Italy; 14 CNR-IASI BioMatLab, Consiglio Nazionale delle Ricerche, Istituto di Analisi dei Sistemi ed Informatica, Laboratorio di Biomatematica (Italian National Research Council, Institute for System Analysis and Computer Science, Biomathematics Laboratory), Rome, Italy; 15 Department of Anesthesia and Intensive Care, Azienda Ospedaliera-Università di Padova, Padua, Italy; 16 Dipartimento di Medicina-DIMED, Università degli Studi di Padova, Via Giustiniani 2, 35128 Padova, Italy

**Keywords:** Acute respiratory distress syndrome, Positive end-expiratory pressure, Mechanical ventilation, Ventilator-induced lung injury

## Abstract

**Background:**

In acute respiratory distress syndrome (ARDS), response to positive end-expiratory pressure (PEEP) is variable according to different degrees of lung recruitability. The search for a tool to individualize PEEP based on patients’ individual response is warranted.

End-expiratory lung volume (EELV) assessment by nitrogen washing-washout aids bedside estimation of PEEP-induced alveolar recruitment and may therefore help titrate PEEP on patient’s individual recruitability.

We designed a randomized trial to test whether an individualized PEEP setting protocol driven by EELV measurement may improve a composite clinical outcome in patients with moderate-to-severe ARDS (IPERPEEP trial).

**Methods:**

IPERPEEP is an open-label, multicenter, randomized trial that will be conducted in 10 intensive care units in Italy and will enroll 132 ARDS patients showing PaO_2_/FiO_2_ ratio ≤ 150 mmHg within 24 h from endotracheal intubation while on mechanical ventilation with PEEP 5 cmH_2_O.

To standardize lung volumes at study initiation, all patients will undergo mechanical ventilation with tidal volume of 6 ml/kg of predicted body weight and PEEP set to obtain a plateau pressure within 28 and 30 cmH_2_O for 30 min (EXPRESS PEEP).

Afterwards, a 5-step decremental PEEP trial will be conducted (EXPRESS PEEP to PEEP 5 cmH_2_O), and EELV will be measured at each step. Recruitment-to-inflation ratio will be calculated for each PEEP range from EELV difference. Patients will be then randomized to receive mechanical ventilation with PEEP set according to the optimal recruitment observed in the PEEP trial (IPERPEEP arm) trial or to achieve a plateau pressure of 28–30 cmH_2_O (control arm, EXPRESS strategy). In both groups, tidal volume size, use of prone positioning and neuromuscular blocking agents, and weaning from PEEP and from mechanical ventilation will be standardized.

The primary endpoint of the study is a composite clinical outcome incorporating in-ICU mortality, 60-day ventilator-free days, and serum interleukin-6 concentration over the course of the initial 72 h of treatment.

**Discussion:**

The IPERPEEP study is a randomized trial powered to elucidate whether an individualized PEEP setting protocol based on bedside assessment of lung recruitability can improve a composite clinical outcome during moderate-to-severe ARDS.

**Trial registration:**

ClinicalTrials.govNCT04012073. Registered 9 July 2019.

**Supplementary Information:**

The online version contains supplementary material available at 10.1186/s13063-021-05993-0.

## Background

Mechanical ventilation is the cornerstone treatment of the acute respiratory distress syndrome (ARDS). However, mechanical ventilation can itself initiate and worsen lung damage due to the so-called ventilator-induced lung injury (VILI), which is an inflammatory response that worsens lung function and contributes to the development of the organ dysfunction observed in ARDS patients [1]. Different strategies have been suggested to attenuate VILI: limiting tidal volume (*V*_T_); plateau and driving pressure mitigates lung injury and improves clinical outcome [2–6].

Low *V*_T_ may generate alveolar derecruitment and worsen oxygenation that can be reversed by the application of a positive end-expiratory pressure (PEEP) [7, 8]. It is widely accepted that PEEP setting should aim to a balance between alveolar recruitment (i.e., re-opening of collapsed alveolar units with increased lung volume available for tidal ventilation) and the damage unavoidably generated in the already open tissue. Hence, over the last decade, great effort has been made to identify the PEEP-setting strategy that balances the need for lung recruitment and PEEP-induced alveolar overdistension; PEEP titration methods based on respiratory system compliance [9–11], oxygenation, shunt value s[12, 13], and pressure-volume curve [14] have been proposed. Four different randomized studies comparing higher versus lower PEEP, with high PEEP set according to respiratory system mechanic s[10], oxygenation impairment [12, 13] or to maximize respiratory system complianc e[15], failed to detect a benefit. Higher PEEP however yielded a shorter time to successful weaning in the EXPRESS study, especially in most severe patients [10].

Despite a meta-analysis highlighted a survival benefit in more severe patients treated with higher PEE P[16], the most relevant drawback of such “universal” strategies is that lung recruitability has great inter-individual variability, with high PEEP enhancing lung injury in patients with low recruitability and low PEEP not fully exerting its beneficial effects in recruitable patients [14, 17–20]. Thus, a search for a tool to individualize PEEP on patient’s individual response appears warranted. In spite of encouraging results coming from a preliminary study [21], neither transpulmonary pressure-guided nor driving pressure-driven PEEP did provide benefits in larger trials [15, 22].

Measurement of end-expiratory lung volume (EELV) by the nitrogen washing-washout technique, bedside available from recent ICU ventilators, has been shown to reliably estimate lung aeration and may help mechanistically titrate PEEP on patient’s individual response [23–25].

We designed an open-label, multicenter, randomized trial to test the safety and feasibility of an individualized PEEP setting protocol driven by EELV and to determine whether this may improve a composite clinical outcome in patients with moderate-to-severe ARDS.

## Methods

### Study design

**Fig. 1:**
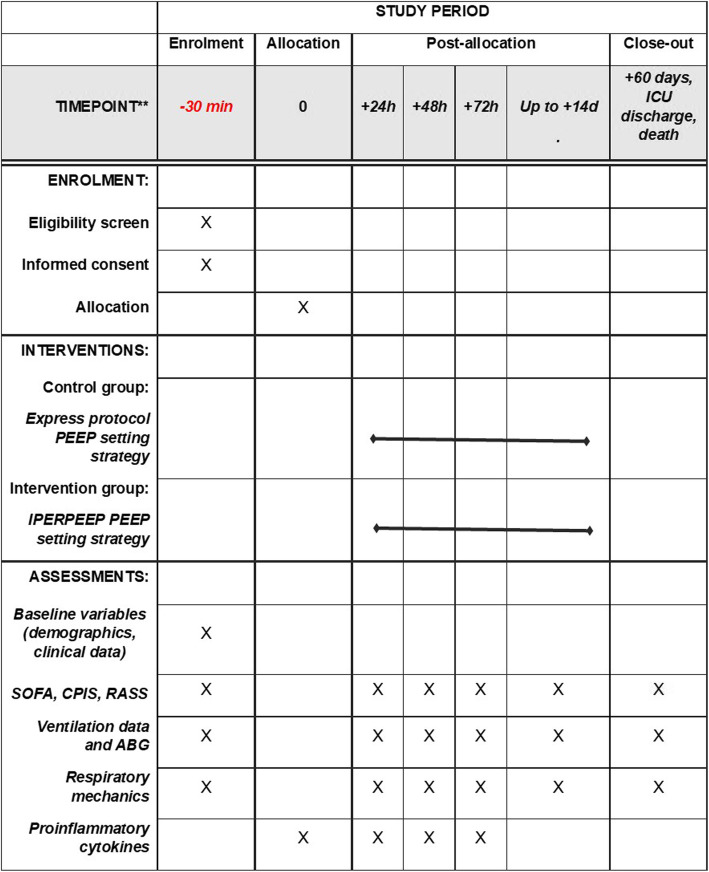
Standard Protocol Items: Recommendation for Interventional Trials (SPIRIT) schedule of enrollment, interventions, and assessments. SOFA, Sequential Organ Failure Assessment; CPIS, clinical pulmonary index score; RASS, Ramsay Agitation Sedation Scale; ICU, intensive care unit

IPERPEEP is an investigator-initiated, multicenter, parallel-group, open-label, two-arm, randomized trial that will be conducted on patients with ARDS and PaO_2_/FiO_2_ ratio equal to or lower than 150 mmHg. The study will be conducted in 10 intensive care units (ICU) in Italy (please refer to Additional file 1 for the details of involved sites). The first patient will be randomized in late 2020. This protocol conforms to the Consolidated Standards of Reporting Trials (CONSORT) guidelines. Figure 1 shows the Standard Protocol Items: Recommendation for Interventional Trials (SPIRIT) schedule of enrollment, interventions, and assessments. By the time of this submission, the protocol has been approved by the ethics committee of the coordinating center (Fondazione Policlinico A. Gemelli IRCCS, ID 38554/18 and 45307/19).

There was no public nor patients’ involvement in the design of the trial.

The study has been registered on clinicaltrials.gov (NCT04012073) on July 9, 2019.

### Objectives of the study

The primary objective of the study is to determine whether a PEEP setting protocol driven by EELV measurement may improve a composite clinical outcome in patients with moderate to severe ARDS, as compared to the EXPRESS protocol [10].

### Selection of the participants

All adult ICU patients requiring invasive mechanical ventilation due to acute hypoxemic respiratory failure will be screened for the enrolment. Patients with ARDS and moderate-to-severe oxygenation impairment (PaO_2_/FiO_2_ ≤ 150 mmHg while receiving controlled mechanical ventilation with PEEP = 5 cmH2O) will be the studied population.

Eligibility inclusion criteria for moderate-to-severe ARDS, according to the Berlin definition, will be assessed within the first 24 h from the initiation of invasive mechanical ventilation [26]:

1. Acute respiratory failure within 1 week of a known clinical insult or new or worsening respiratory symptoms;
2. Bilateral infiltrates at the chest x-ray or CT scan, not fully explained by effusions, lobar/lung collapse, or nodules;
3. Respiratory failure not fully explained by cardiac failure or fluid overload; objective assessment required to exclude hydrostatic edema if no risk factor present.
4. PaO_2_/FiO_2_ ratio ≤ 20 0[27].
5. Written informed consent.

Exclusion criteria will be as follows: pregnancy, pneumothorax, acute brain injury, decompensated heart failure (NYHA 3–4 before the acute phase of the disease or documented ejection fraction< 35% or pulmonary capillary wedge pressure > 18 mmHg) or acute coronary syndrome, intubation as a result of an acute exacerbation of chronic pulmonary disease, body mass index greater than 35 kg/m^2^ or lower than < 35 kg/m^2^, any chronic disease requiring long-term oxygen therapy or mechanical ventilation at home, neuromuscular diseases, severe chronic liver disease (Child-Pugh C or worse), bone marrow transplantation or chemotherapy-induced neutropenia, history of liver or lung transplant, decision to withhold life-sustaining treatment, need for therapy with inhaled nitric oxide due to documented pulmonary arterial hypertension, life-threatening hypoxemia deemed to require extracorporeal membrane oxygenation (ECMO), documented barotrauma, high risk of mortality within 3 months from other than ARDS (severe neurological damage, age > 85 years and cancer patients in terminal stage of the disease), and persistent hemodynamic instability/intractable shock (norepinephrine > 1 mcg/kg/h and/or blood lactate > 5 mmol/L and/or considered too hemodynamically unstable for enrolment in the study by the attending physician).

### Oxygenation criterion validation

Each patient meeting inclusion criteria will be evaluated for the presence of the oxygenation criterion. After neuromuscular paralysis and endotracheal suctioning, eligible patients will be ventilated for 30 min with PEEP = 5 cmH_2_O in the semi-recumbent position; afterward, arterial blood gas analysis (ABG) will be performed to compute PaO_2_/FiO_2_ ratio. Patients showing PaO_2_/FiO_2_ ≤ 150 mmHg will be enrolled. Patients showing PaO_2_/FiO_2_ < 200 and > 150 mmHg will be treated according to the standard clinical practice and reassessed for the presence of oxygenation criterion within 24 h from endotracheal intubation. To limit the exposure to low PEEP of possibly recruitable patients with severe oxygenation impairment, the ABG certifying the oxygenation criterion will be permitted at any time during the 30-min monitoring period.

### Procedures

After inclusion in the study, all patients will receive continuous neuro-muscular blocking agents (NMBA) infusion for 48 h and will be connected to a mechanical ventilator equipped with EELV measurement module (Carescape R860, GE Healthcare, USA). A standard bi-tube low-resistance circuit with a low-dead space, low-resistance, and high-efficiency heat and moisture exchanger or a heated and humidified bi-tube circuit will be used to connect the patient to the ventilator, according to the preference of the attending physician and the practice of each institution. The use of heated and humidified bi-tube circuits (advised settings: humidification chamber temperature set at 37 °C, absolute humidity provided 44 mgH_2_O/L) will be encouraged in patients that remain hypercapnic (pH < 7.30 and PaCO_2_ > 50) despite all ventilator settings provided by the study protocol [28].

A dedicated polyfunctional feeding tube provided with an esophageal balloon (Nutrivent, Sidam, Mirandola (MO), Italy) to monitor esophageal pressure, estimate pleural pressure, and compute transpulmonary pressure will be placed in all enrolled patients after inclusion. The correct placement of the catheter and the adequate filling of the balloon will be confirmed by an occlusion test (airway pressure/esophageal pressure ratio will be recorded: 0.8–1.2 will be considered acceptable); the occlusion test will be repeated before any measurement is done to ensure the recordings of reliable values [29, 30].

A closed endotracheal suctioning system will be preferentially used in all the enrolled patients.

After the enrollment, mechanical ventilator will be set according to the following strategy: *V*_T_ = 6 mL/kg of predicted body weight; inspiratory flow set at 60 l/min resulting in an end-inspiratory pause of 0.3–0.5 s; respiratory rate 20–35 to maintain pH > 7.30, PEEP set so that the plateau (*P*_PLAT_) pressure will be within the following limits: *P*_PLAT_ = 28–30 cmH_2_O (EXPRESS PEEP), FiO_2_ set to achieve a SpO_2_ > 88–95% and not higher than 90%. In case of hypercapnia with pH < 7.30 despite a respiratory rate = 30–35, an increase in *V*_T_ up to 8 ml/kg will be allowed over the entire course of the study.

Predicted body weight (PBW) will be calculated according to the following formulas [3]:

$$ \mathrm{Males}:\mathrm{PBW}\ \left(\mathrm{kg}\right)=50+0.91\ \left(\mathrm{height}\ \mathrm{in}\ \mathrm{cm}-152.4\right) $$

$$ \mathrm{Females}:\mathrm{PBW}\ \left(\mathrm{kg}\right)=45.5+0.91\ \left(\mathrm{height}\ \mathrm{in}\ \mathrm{cm}-152.4\right) $$

After 30 min with these settings to standardize lung volumes at maximum recruitment, a new ABG will be recorded. Afterwards, a 5-step decremental PEEP trial (EXPRESS PEEP to PEEP 5) will be conducted with the dedicated software on the ventilator (Inview PEEP, GE). Each step will last 8 min, and EELV will be measured with a 0.2 change in the FiO_2_. In case of evident airway closure [31, 32], the minimal PEEP tested in the PEEP trial will be the one providing a PEEP_TOT_ = airway opening pressure [32].

PEEP-induced lung recruitment across each PEEP step (Rec) will be computed as follows:

23 $$ \mathrm{Rec}=\left({\mathrm{EELV}}_{\mathrm{PEEP}\mathrm{high}}-{\mathrm{EELV}}_{\mathrm{PEEP}\mathrm{low}}\right)-\left[\left({\mathrm{PEEP}}_{\mathrm{high}}-{\mathrm{PEEP}}_{\mathrm{low}}\right)\times {\mathrm{Compliance}}_{\mathrm{PEEP}\mathrm{low}}\right] $$

After the PEEP trial, a one-breath derecruitment maneuver from PEEP 5 to PEEP 0 will be conducted to assess baseline functional residual capacity (FRC) that will be measured as the difference between EELV at PEEP 5 and the lung volume increase above FRC, measured as the difference in expired tidal volume as PEEP is decreased from 5 to 0 cmH_2_O in one breath with respiratory rate ≤ 8 breaths per minute. In particular, the lung volume due to the presence of PEEP 5 (PEEP5_volume_) will be measured by subtracting the insufflated *V*_T_ from the expired *V*_T_ during a 5-s exhalation (respiratory rate < 8) just after PEEP is reduced from 5 to 0.

$$ \mathrm{FRC}={\mathrm{EELV}}_{\mathrm{PEEP}5}-\mathrm{PEEP}{5}_{\mathrm{volulme}} $$

This procedure will not be conducted in patients with airway closure.

In particular, when interpreting the results of the PEEP trial, Rec across two adjacent PEEP levels will be normalized to the changes in the effective PEEP (total PEEP) applied: REC_IND_/cmH_2_O will be computed as REC divided by the PEEP difference. For each PEEP range, the recruitment-to-inflation ratio (RI) will be then calculated as previously described [19, 33]:

$$ \mathrm{RI}={\mathrm{REC}}_{\mathrm{IND}}/{\mathrm{Compliance}}_{\mathrm{PEEPlow}}. $$

Each patient will be then randomized to undergo mechanical ventilation according to the two PEEP-setting protocols described in the next paragraph. Randomization will be stratified according to the volume of effective lung recruitment during the PEEP trial, as described in the “Randomization and record keeping” section.

Patients for whom the measurement of the EELV is not be feasible due to any technical reason will not be randomized and accurately recorded on the screening log.

### PEEP setting protocols

#### Control group

The EXPRESS protocol is as follows [10]: *V*_T_ = 6 mL/kg; inspiratory flow set at 60 l/min and end-inspiratory pause of 0.2-0.5 sec, PEEP set so that *P*_PLAT_, measured during the end-inspiratory pause of 1–3 s, will be within the following limits: 28 cmH_2_O ≤ *P*_PLAT_ ≤ 30 cmH_2_O;. FiO_2_ set to achieve a SpO_2_ > 88–95%.

In case of hypercapnia with pH < 7.30 despite a respiratory rate = 30–35, an increase in *V*_T_ up to 8 ml/kg will be allowed.

#### Intervention group

**Fig. 2:**
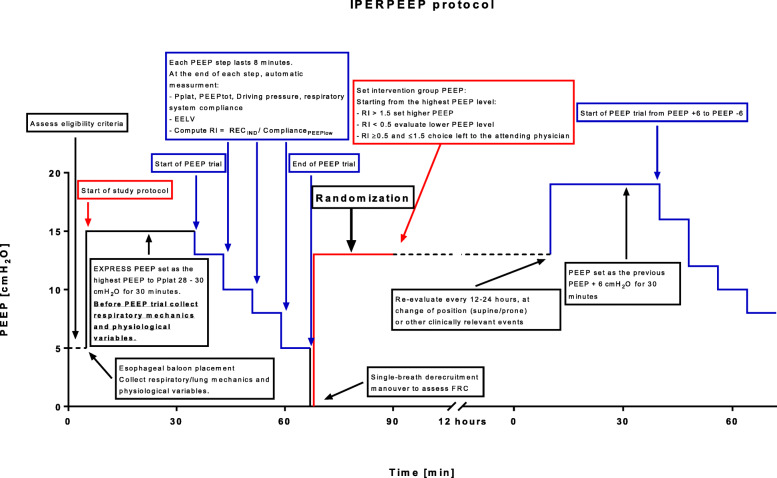
Graphical representation of study procedures before randomization and after randomization in the IPERPEEP arm

Full details of the procedures performed in the intervention group are provided in Fig. 2.

*V*_T_ = 6 mL/kg; inspiratory flow set at 60 l/min resulting in an end-inspiratory pause of 0.2–0.5 s. Respiratory rate set between 20 and 35 to maintain pH > 7.30. FiO_2_ set to achieve a SpO_2_ > 88–95%.

In case of hypercapnia with pH < 7.30 despite a respiratory rate = 30–35, an increase in VT up to 8 ml/kg will be allowed.

PEEP will be ≥ 5 cmH_2_O and set to ensure the maximum recruitment observed in the PEEP trial, with a maximum permitted *P*_PLAT_ of 30 cmH_2_O.

In particular, when interpreting the results of the PEEP trial, RI across two adjacent PEEP levels will drive PEEP setting.

- RI ≥ 1.5 between two PEEP levels will lead to the setting of the higher PEEP.
- RI < 0.5 will lead to the setting of the lower PEEP value.
- In case of RI ≥ 0.5 and < 1.5, the choice among two adjacent PEEP levels will be left to the attending physician, who will indicate the set PEEP in order to best balance between the commitment of limiting total, static, and dynamic strain and of optimizing oxygenation and hemodynamics.

Due to possible interferences with EELV measurement, in patients showing airway closure [31, 32, 34], no PEEP lower than the airway opening pressure will be tested or used in the IPERPEEP group for the whole duration of the intervention.

A similar 5-step PEEP trial will re-assess lung response to different levels of PEEP every 12–24 h, in case of changes in the body position or any other clinical events requiring modifications of the ventilatory settings.

Following PEEP trials will be conducted to investigate RI at the five PEEP levels closest to the set one.

Before the procedure, PEEP will be set for 15 min at the maximum PEEP expected to be tested during the PEEP trial, to ensure that *P*_PLAT_ remains below the safety limit with this setting: no PEEP< 5 cmH2O, generating *P*_PLAT_ > 30 cmH_2_O or lower than the airway opening pressure (in case of airway closure) will be tested or used over the entire course of the study in this intervention group. In particular, each following PEEP trial will investigate response to PEEP at values ranging from + 6 and − 6 than the previously set one (i.e., in case of previously set PEEP = 12, PEEP trial at 6-9-12-15-18; in case of previously set PEEP = 16, PEEP trial at PEEP = 10-13-16-19-22; in case of previously set PEEP = 8, PEEP trial at PEEP = 5-8-11-14).

The PEEP level will be then modified according to the strategy described above.

In both groups, the assigned ventilation protocol will be followed for a minimum of 72 h from randomization and any time fully controlled ventilation is deemed necessary by the attending physician up to 14 days from randomization. After 14 days from randomization, PEEP will be set according to the clinical practice of each institution.

### Measurements

All patients intubated due to hypoxemic respiratory failure and not included will be recorded on a dedicated screening log.

Demographics will be collected at study entry. Physiological variables will be recorded at the moment of enrolment (PEEP 5), after maximum PEEP is set before the PEEP trial, 1-6-12-24-48 h after randomization, and then on a daily basis up to 60 days or ICU discharge.

Data of the ventilation management (type of ventilation, FiO_2_, tidal volume, mean airway pressure, PEEP, plateau pressure, minute ventilation, respiratory rate, esophageal end-inspiratory and end-expiratory pressures, static stress, dynamic stress and total stress, proportion of spontaneous ventilation on minute ventilation, VO_2_, VCO_2_), physiologic data (heart rate, arterial pressure, SpO_2_, end-tidal CO_2_), and results of the blood gas analyses (pH, SaO_2_, PaO_2_, PaCO_2_, SvO_2_, PvCO_2_, oxygenation index, dead space fraction) will be collected.

*Respiratory mechanics* will be recorded at the moment of enrolment (PEEP 5), at each step of the first PEEP trial, and 1-6-12-24-48 h from randomization and then on a daily basis (72-96-120…) up to 60 days or ICU discharge. End-expiratory airway pressure (PEEP_AW_) and the end-expiratory esophageal pressure (PEEP_ES_) will be recorded during a 4-s expiratory hold. End-inspiratory airway pressure (*P*plat_AW_) and end-inspiratory esophageal pressure (*P*plat_ES_) will be measured during a 1-s end inspiratory hold. Expired tidal volume (*V*_T_) will be measured as the integration of the flow-time curve during expiration.

End-expiratory airway pressure (PEEP_AW_) and the end-expiratory esophageal pressure (PEEP_ES_) will be recorded during a 4-s expiratory hold. End-inspiratory airway pressure (*P*plat_AW_) and end-inspiratory esophageal pressure (*P*plat_ES_) will be measured during a 1-s end inspiratory hold. Expired tidal volume (*V*_T_) will be measured as the integration of the flow-time curve during expiration.

Advanced respiratory mechanics parameters will be computed afterwards. Stress will be calculated any time respiratory mechanics is measured, while strain will be measured before randomization in all patients and in the intervention group any time a PEEP trial is repeated; formulas are also shown in Table 1.

**Table 1:** Computed respiratory mechanics parameters

*Parameter*	*Formula*
Airway driving pressure	ΔP = *P*plat_aw_ − PEEP_aw_
Transpulmonary end-inspiratory pressure	*P*plat_L_ = Plat_aw_ − *P*plat_es_
Transpulmonary end-expiratory pressure	PEEP_L_ = PEEP_aw_ − PEEP_es_
Lung driving pressure	ΔP_L_ = *P*plat_L_ − PEEP_L_
Lung plateau pressure, elastance-derived	*P*plat_L,EL_ = *P*plat_aw_ × (ΔP_L_/ΔP)
Static respiratory system compliance	Cst_RS_ = *V*_T_/ΔP
Static lung compliance	Cst_L_ = *V*_T_/ΔP_L_
Static chest wall compliance	Cst_CW_ = *V*_T_/(*P*plat_es_ − PEEP_es_)
Oxygenation-stretch index	OSI = PaO_2_/(FiO_2_ × ΔP)
Dynamic strain	Dynamic strain = *V*_T_/FRC_PEEPset_
Static strain (strain due to PEEP)	Static strain = (PEEP_volume_ − Rec_ZEEP-PEEP_)/FRC_PEEPset_
Static stress (stress due to PEEP)	Static stress = PEEP_aw_ × (ΔP_L_/ΔP)
Dynamic stress	Dynamic stress = *P*plat_aw_ × (ΔP_L_/ΔP)

The levels of *proinflammatory cytokines* (tumor necrosis factor [TNF], interleukin 6 [IL-6], and interleukin 8 [IL-8]) will be assessed from serum probes (enzyme-linked immunosorbent assay (ELISA)) of all included patients at study inclusion and 24, 48, and 72 h after randomization.

The following *scoring systems* will be calculated daily: simplified organ failure assessment score (SOFA), modified clinical pulmonary infection score (CPIS), and Richmond agitation and sedation scale (RASS). These systems and organ failure definitions used for the study purposes are reported in Additional file 2.

### Concomitant patient medical management

Concomitant patients’ management, including medical treatments and procedures, is detailed in Additional file 3.

### Study endpoints

The primary endpoint will be a composite clinical outcome that incorporates in-ICU mortality, 60-day ventilation-free days (VFD60), and the area under the curve of the interleukin-6 serum blood cytokine concentration (IL6AUC) during the first 72 h of observation.

Secondary endpoints will be as follows: ICU-, 90-day and in hospital mortality, 28 and 60-day ventilation-free days (VFD60), the area under the curve (AUC) of serum blood cytokines concentrations measured as stated in the study protocol; lung static, dynamic and total stress and strain after randomization; airway and lung driving and plateau pressures, compliance, compliance/PBW, dead space fraction, PaO_2_/FiO_2_ ratio, oxygenation stretch index and oxygenation index during the study treatment; and SOFA, CPIS, the incidence of complications or adverse reactions, the frequency and duration of other adjunctive therapeutic measures, transfusion requirements, the daily cumulative doses of analgesic and sedative agents, cumulative catecholamine requirements/24 h throughout the study period, frequency and duration of renal replacement therapy, the number of failing organs, and the “organ-failure-free days” within 28 days after randomization.

### Randomization and record keeping

Patient data, clinical status, laboratory results, and respiratory parameters will be collected on a web-based case report form (Ferrariodati, Italia). Participants will be randomized in a ratio of 1:1. Randomization will be performed by an electronic, web-based centralized, validated system.

Randomization will be stratified according to:

- RI ≥ 1 and < 1 across the range between the lowest and highest PEEP tested during the PEEP trial [23] (see the “Procedures” section for details).

All original records will be archived and secured for 10 years and then destroyed according to the hospital standards concerning destruction of confidential information.

### Data monitoring

Data monitoring will be performed for quality control purposes by an independent company not involved in the study (Clinical Trial Center, Rome, Italy). Amis of monitoring will be to evaluate the study progress and to verify the accuracy of data recording.

### Statistics

All collected data will be tabulated descriptively by study group and analyzed on an intention-to-treat basis. Comparisons between groups will be performed with the chi-squared test or the Fisher’s exact test, as appropriate. Ordinal qualitative variables or non-normal quantitative variables will be compared with the Wilcoxon sum of ranks test. Quantitative normal variables will be compared with the Student *t*-test. In particular, analysis of the primary efficacy criterion will be performed with Wilcoxon-Mann-Whitney sum of ranks test.

Consistently to other published studies [22], every participant in the treatment group will be compared with every participant in the control group and assigned a score resulting from each comparison.

Mortality takes precedence over VFD60, which takes precedence over IL6AUC. Two VFD60s will be considered different for the purpose of scoring only if their difference is larger than 5 days. Similarly, two IL6AUC’s measurements will be considered different only if their difference exceeds 10% of the smaller of the two.

These individual-comparison scores are added up to obtain the cumulative score primary endpoint for each participant. Scoring system for the primary endpoint is detailed in Additional file 4.

For the purpose of the study, mechanical ventilation will be defined as: invasive mechanical ventilation with endotracheal tube or tracheostomy and noninvasive positive pressure ventilation, including continuous positive airway pressure. High flow oxygen therapy through nasal cannula or tracheostomy will not be considered as mechanical ventilation.

The assumptions and methodology used for the determination of the sample size are detailed in Additional file 5 [35]. Given the results obtained, and adopting a conservative approach in order to compensate for the small residual random variation in the numerical assessment of the sample size, it appears that a sample size of 56 subjects per arm would be sufficient to obtain 80% power in detecting a difference in the composite endpoint, at a type 1 error level of 0.05, two-tail. With an attrition rate of 15%, a total of 132 patients in two equal groups of 66 patients each should be enrolled. This corresponds to a mean of 19 patients per center.

Primary and secondary endpoints will also be analyzed in subgroups, as defined below:

- ∆EELV5-16/FRC ≥ 73% [18] during the PEEP trial
- ∆EELV5-16/FRC < 73 %[23] during the PEEP trial
- RI ≥ 1 and < 1 across the range between the lowest and highest PEEP tested during the PEEP trial
- P/F ratio < 100 mmHg at study inclusion
- IL-6 > 400 pg/ml at study inclusion

All analyses will be performed at a 0.05 type I error level, two tail. All statistical analyses on secondary endpoints and on subgroups will be deemed to be exploratory in nature; for this reason, no correction of nominal significance levels for multiple inference will be applied.

### Safety/feasibility analysis, adverse events, and interruption of the trial

A Safety Monitoring Committee (SMC) will conduct an analysis to assess the safety and feasibility of the IPERPEEP protocol after 30 patients complete the study, and the study will be continued only if both safety and feasibility are established. In this assessment, no other data besides the criteria for safety and feasibility will be unblinded. The study will be considered feasible if at least 80% of the patients randomized to the intervention group will correctly undergo the allocated treatment according to the study protocol. The study will be considered safe if no serious adverse events related to treatment will be detected in the intervention group while the allocated treatment is ongoing.

Serious adverse events (SAE) will be defined as any event that is fatal or immediately life threatening, permanently disabling, severely incapacitating, or requires prolonged hospitalization or that may jeopardize the patient and requires medical or surgical intervention to prevent one of the outcomes listed above. SAE will be considered related to the treatment if the attending physician believes that they might be directly related to enrollment in the clinical trial. In particular, SAE’s will be considered to be study-related if the event follows a study procedure and could readily have been produced by the study procedure.

## Discussion

This investigator-initiated, pragmatic, multicenter, prospective, interventional, open-label randomized clinical trial will assess the potential benefits for ARDS patients of a PEEP-setting strategy based on individual patients’ response to PEEP in terms of alveolar recruitment and overdistension, as assessed by sequential measurement of EELV by the nitrogen washing-washout technique during a PEEP trial. Indeed, alveolar recruitability as a response to PEEP has wide inter-subject variability, and it is well established that PEEP can increase overdistension and thus static strain, while decreasing dynamic strain only if recruiting new alveolar units for tidal ventilations [36]. This variability in the response to PEEP between patients may explain the failure of several recent randomized trial to demonstrate a universal benefit from an overall high-PEEP strategy [10, 12, 13], calling for the need of individualized strategies based on the actual measurement of recruitment and overdistension [20]. Such a strategy could be feasible with this bedside technique, which has been shown to reliably estimated lung’s aerated volume [23–25] and may overcome the shortcomings of other recently proposed physiology-based PEEP titration techniques [9–14]. The potential implications of the results from this well-powered physiological multicenter trial could lead to an improvement in the knowledge on individualized PEEP titration in ARDS and may improve future ventilatory management of these patients.

### Trial status

Protocol version 2.0, July 2019.

At the time of submission, the regulatory authorization has been obtained in the coordinating center. Enrolment has not yet started in the participating centers. First inclusion is planned in November 2022, and completion of recruitment is estimated to be completed in December 2024.

## Supplementary Information

**Additional file 1:** Participating centers.

**Additional file 2:** Organ failure definitions and scoring systems.

**Additional file 3:** Concomitant patient medical management.

**Additional file 4:** Scoring system for the primary endpoint.

**Additional file 5:** Sample size determination: assumptions and methodology.

## Data Availability

Not applicable

## Abbreviations

ARDS: Acute respiratory distress syndrome
AOP: Airway opening pressure
BMI: Body mass index
CNS: Central nervous system
COPD: Chronic obstructive pulmonary disease
CRF: Case report form
CPIS: Modified clinical pulmonary infection score
Cst_L\_: Static lung compliance
Cst_RS_: Static respiratory system compliance
Crec: Compliance of the recruited lung
CT scan: Computed tomography scan
ECCO_2_-R: Extracorporeal CO_2_ removal
ECMO: Extracorporeal membrane oxygenation
EELV: End-expiratory lung volume
EELV_REC_: Recruited volume
ELISA: Enzyme-linked immunosorbent assay
FiO_2_: Fraction of inspired oxygen
FRC: Functional residual capacity
FRC_PEEPset_: Aerated volume at set PEEP
GE Healthcare: General Electric Healthcare
ICU: Intensive care unit
ICUdeath: Mortality in ICU
IL: Interleukin
IL6AUC: Area under the curve of the interleukin-6 serum blood cytokine concentration
NIV: Noninvasive ventilation
NMBA: Neuromuscular blockade
NYHA: New York Heart Association
PBW: Predicted body weight
PaO_2_: Arterial oxygen partial pressure
PEEP: Positive end-expiratory pressure
PEEP_AW_: End-expiratory airway pressure
PEEP_ES_: End-expiratory esophageal pressure
PEEP_L_: End-expiratory transpulmonary pressure
PEEP_ES,ZEEP_: End-expiratory esophageal pressure
*P*plat_AW_: End-inspiratory airway pressure
*P*plat_ES_: End-inspiratory esophageal pressure
*P*plat_ESO_: End-inspiratory esophageal pressure
*P*plat_L,EL_: End-inspiratory elastance-derived transpulmonary pressure
*P*plat_L_: Transpulmonary pressure
RASS: Richmond Agitation and Sedation Scale
Rec_ZEEP-PEEP_: Recruited volume from ZEEP to PEEP
R/I: Recruitment-to-inflation ratio
SAPSII: Simplified Acute Physiology Score
SBT: Spontaneous breathing trial
SOFA: Sequential Organ Failure Assessment
SpO_2_: Oxygen peripheral saturation
TNF: Tumor necrosis factor
*P*_PLAT_: Plateau pressure
VFD60: 60-day ventilation-free days
VILI: Ventilator-induced lung injury
*V*_T_: Tidal volume
