# Supplementary material for: Individualized positive end-expiratory pressure guided by end-expiratory lung volume in early acute respiratory distress syndrome: study protocol for the multicenter, randomized IPERPEEP trial
Source: Trials. 2022 Jan 20;23:63. doi: 10.1186/s13063-021-05993-0 (PMC8772175; doi:10.1186/s13063-021-05993-0)
Supplement: Supplementary file 1 — Additional file 1: Participating centers. [file 13063_2021_5993_MOESM1_ESM.docx]

**Additional file 1:** Participating centers

| **CITY** | **NAME OF THE INSTITUTION** | **NAME OF THE OPERATIVE UNIT** | **NAME OF THE PI** |
| --- | --- | --- | --- |
| Rome | Fondazione Policlinico Universitario A. Gemelli IRCCS | Rianimazione, Terapia Intensiva e Tossicologia Clinica | Massimo Antonelli |
| Bologna | Policlinico S.Orsola-Malpighi | Anestesia e Terapia intensiva Polivalente | Elisabetta Pierucci |
| Chieti | Policlinico «SS. Annunziata» | Anestesia, rianimazione e terapia intensiva | Salvatore Maurizio Maggiore |
| Pavia | Fondazione IRCCS Policlinico San Matteo | Anestesia e Rianimazione I | Francesco Mojoli |
| Monza | Ospedale San Gerardo | Terapia Intensiva generale | Giuseppe Foti |
| Bari | Azienda Ospedaliero Universitaria Consorziale Policlinico | Rianimazione II | Salvatore Grasso |
| Catanzaro | Azienda Ospedaliero-Universitatia Mater Domini | Anestesia e rianimazione | Federico Longhini |
| Milano | Fondazione IRCCS Ca’ Granda-Ospedale Maggiore Policlinico | Rianimazione e terapia intensiva adulti | Giacomo Grasselli |
| Genova | Ospedale Policlinico San Martino IRCCS | Clinica anestesiologica e terapia intensiva | Paolo Pelosi |
|  |  |  |  |
| Ferrara | Arcispedale Sant’Anna | Anestesia e Rianimazione Universitaria | Carlo Alberto Volta |
