# Supplementary material for: Individualized positive end-expiratory pressure guided by end-expiratory lung volume in early acute respiratory distress syndrome: study protocol for the multicenter, randomized IPERPEEP trial
Source: Trials. 2022 Jan 20;23:63. doi: 10.1186/s13063-021-05993-0 (PMC8772175; doi:10.1186/s13063-021-05993-0)

**Additional file 2:** Organ failure definitions and scoring systems

**Organ failure definitions**

• Sepsis: Two or more of the following: 1) a rectal or core temperature > 39C; 2) a total leukocyte count higher than 12,000/mm3 or with more than 20% immature forms; 3) a blood culture positive for a recognized pathogen; 4) gross pus in a closed space, or 5) a positive culture from a known or strongly suspected source of systemic infection. In addition, the diagnosis requires any of the following systemic responses: 1) unexplained systemic arterial hypotension (< 85mmHg systolic) for more than 2 hours; 2) systemic vascular resistance of less than 800 dynes/s/cm5, or 3) unexplained metabolic acidosis.

• Pulmonary aspiration: Recent inhalation of gastric contents, documented by suctioning gastric contents from the endotracheal tube in a patient at high risk for aspiration.

• Coma: Diminished level of consciousness with a Glasgow coma scale < 7 in the absence of therapeutic coma.

• Acute heart failure: A clinical picture of left ventricular failure or a pulmonary capillary wedge pressure more than 18 mmHg and a low cardiac output.

• Acute renal failure: Defined by a serum creatinine level of > 2 mg/dL. In patients with pre-existing renal disease, doubling of the admission creatinine level is considered an indicator of acute renal failure.

• Disseminated intravascular coagulation: A platelet count less than 50,000/mm3, elevated fibrin degradation products, and a fibrinogen level less than 200 mg/dl with or without clinical evidence of spontaneous bleeding.

• Hepatic failure: A serum bilirubin level greater than 2 mg/dL with elevation of the transaminase and lactic dehydrogenase levels above twice normal values.

• Acute gastrointestinal tract failure: Gastrointestinal bleeding requiring two or more units of blood replacement therapy within a 24-hour period.

• Shock: A systemic systolic blood pressure below 85 mmHg for over 2 hours with evidence of organ hypoperfusion, or a systolic blood pressure of 85 mmHg or more maintained by vasopressors.

**Scoring systems**

**Sequential Organ Failure Assessment (SOFA) score**

| **SOFA score** | **0** | **1** | **2** | **3** | **4** |
| --- | --- | --- | --- | --- | --- |
| **Respiration** PaO_2_/FIO_2_ (mmHg) | >400 | <400 | <300 | <200 | <100 |
| **Coagulation** Platelets 10^3^/mm^3^ | >150 | <150 | <100 | <50 | <20 |
| **Liver** Bilirubin (mg/dL) | <1.2 | 1.2–1.9 | 2.0–5.9 | 6.0–11.9 | >12.0 |
| **Cardiovascular^b^** Hypotension | No hypotension | MAP <70 | Dopamine ≤5 or dobutamine (any) | Dopamine>5 or norepinephrine≤0.1 | Dopamine>15 or norepinephrine>0.1 |
| **CNS** Glasgow Coma Score | 15 | 13–14 | 10–12 | 6–9 | <6 |
| **Renal** Creatinine (mg/dL) or urine output (mL/d) | <1.2 | 1.2–1.9 | 2.0–3.4 | 3.5–4.9  or  <500 | >5.0  or  <200 |

**Modified Clinical Pulmonary Infection score (CPIS)**

| **CPIS Points** | **0** | **1** | **2** |
| --- | --- | --- | --- |
| ***Tracheal secretions*** | Rare | Abundant | Abundant + purulent |
| ***Chest X-ray infiltrates*** | No infiltrate | Diffused | Localized |
| ***Temperature, °C*** | ≥ 36.5 and ≤ 38.4 | ≥ 38.5 and ≤ 38.9 | ≥ 39 or ≤ 36 |
| ***Leukocytes count, per mm^3^*** | ≥ 4,000 and ≤ 11,000 | < 4,000 or > 11,000 | < 4,000 or > 11,000  + band forms ≥ 500 |
| ***PAO2/FIO2, mm Hg*** | > 240 or ARDS |  | ≤ 240 and no evidence of ARDS |
| ***Microbiology*** | Negative |  | Positive |

**Richmond Agitation Sedation Scale (RASS score)**


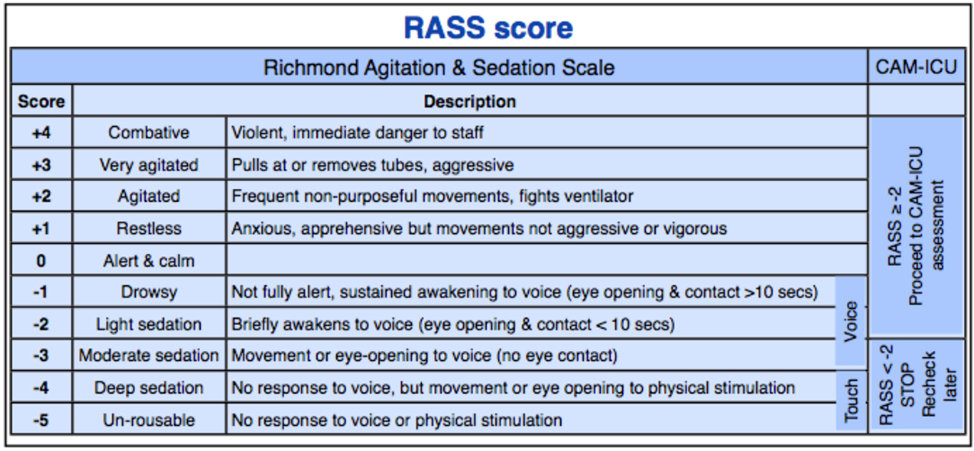

Supplement: Supplementary file 2 — Additional file 2: Organ failure definitions and scoring systems. [file 13063_2021_5993_MOESM2_ESM.docx]
