# Supplementary material for: Individualized positive end-expiratory pressure guided by end-expiratory lung volume in early acute respiratory distress syndrome: study protocol for the multicenter, randomized IPERPEEP trial
Source: Trials. 2022 Jan 20;23:63. doi: 10.1186/s13063-021-05993-0 (PMC8772175; doi:10.1186/s13063-021-05993-0)
Supplement: Supplementary file 3 — Additional file 3: Concomitant patient medical management. [file 13063_2021_5993_MOESM3_ESM.docx]

**Additional file 3:** Concomitant patient medical management

***Standard Care***

In both arms, standard care (diagnostic tests, antibiotics administration ecc.) will be applied according the decision of the attending physician and the practice of each institution.

***Neuromuscolar blockade***

All patients will receive NMBA for 48 hours after the enrolment. The decision to stop NMBA administration after 48 hours will be left to the attending physician, but muscle paralysis will be strongly encouraged if PaO_2_/FiO_2_ ratio remains lower than 80-100 mmHg. NMBA administration will be resumed anytime deemed necessary by the attending physician.

***Endotracheal suctioning***

Endotracheal suctioning will be performed at study screening (before the validation of the oxygenation criterion), after changes in body position and any other time deemed clinically indicated by the attending physician. Routine endotracheal suctioning will be avoided over the entire course of the study. The number of endotracheal suctioning procedures/day in the period when PEEP is set according to the study protocol will be recorded.

In the intervention group, after an endotracheal suctioning is performed with a closed system, a minimum of 5 minutes will be waited before initiating a PEEP trial. On the contrary, in case of open suctioning or any other disconnection from the ventilator, 30 minutes will be waited before conducting a PEEP trial[23,24].

***Prone position***

Prone positioning will be used in all enrolled patients as a standard of care: the decision about the timing and the duration of prone position sessions will be left to the attending physician and the time spent by the patient in the prone and in the supine position will be recorded: PEEP will be re-set according to the protocol of the allocated treatment anytime patient’s position is changed.

***Hemodynamic management***

Overall fluid management guidelines should conform to the Surviving Sepsis Campaign guidelines.

Fluid (crystalloids) will be initially used to maintain:

• Mean arterial pressure > 65 mmHg

• Urine output > 0.5 mL/kg/h

• Pulse pressure variation < 10%

Albumin use will be allowed to maintain serum albumin>20-30 g/L.

Fluid overload will be discouraged. If appropriate fluid challenge fails to restore adequate blood pressure and organ perfusion and if the patient is deemed not to be fluid-responsive, therapy with vasopressor agents will be started.

In case of severe haemodynamic instability, cardiac arrest, pneumothorax or any other adverse event possibly related or worsened by ventilator settings, PEEP reduction will be allowed in both groups if considered necessary by the attending physician: the adverse event will be recorded in the CRF and the assigned treatment will be resumed after the stabilization phase.

Norepinephrine will be the first-choice vasopressor agent to correct hypotension in septic shock.

Vasopressin use may be considered in patients with refractory shock despite adequate fluid resuscitation and high-dose conventional vasopressors.

In patients with low cardiac output despite adequate fluid resuscitation, dobutamine may be used to increase cardiac output. If used in the presence of low blood pressure, it should be combined with vasopressor therapy.

***Nutrition/Glucose Control***

Enteral nutrition should be provided as soon as it is deemed safe by the treating physicians. Exogenous insulin should be provided with the goal a blood glucose level below 180-200 mg/dl.

***Rescue therapies***

Recruitment maneuvers, extracorporeal membrane oxygenation (ECMO), extracorporeal CO_2_ removal (ECCO_2_-R) after randomization will be allowed in both groups as rescue therapies and according to the decision of attending physicians: any of these procedures will be accurately recorded on the CRF.

During ECMO, patients will be initially managed with volume-control ventilation: tidal volume will be 2-4 ml/kg PBW and respiratory rate will be set between 5 and 15 breaths per minute.

In the control group, PEEP will be set in order to achieve a plateau pressure of 22-25 cmH_2_O.

In the intervention group, a pediatric airway module for EELV measurement (GE healthcare) will be used and PEEP will be set as indicated by the intervention protocol. However, if the very low tidal volumes used during ECMO happen to hamper EELV measurement, a PEEP slightly higher (+ 2-4 cmH2O, aiming at balancing the derecruitment caused by ultraprotective ventilation) than the value set according to the treatment protocol before the initiation of ECMO will be suggested in the intervention group.

In both groups, set PEEP during ECMO initiation will not be lower than the one set during standard mechanical ventilation.

During ECMO, the assigned treatment will be carried on while the patient is managed with fully controlled ventilation (defined as the absence of oesophageal pressure inspiratory swings during a 15-second expiratory pause): in the presence of spontaneous breathing, PEEP will be set according to the decision of the attending physician.

However, in order to standardize the treatments, moderate PEEP (10-15 cmH2O) will be encouraged in control group, while PEEP close to the value set during controlled ventilation according to the treatment protocol but <15 cmH2O will be suggested in the intervention group.

During assist/control and assist ventilation, PEEP will never be higher than the last PEEP set according to the assigned protocol during controlled ventilation.

The choice about the timing of spontaneous breathing initiation and weaning from ECMO will be left to the attending physician.

The PEEP-setting protocol according to the assigned treatment will be resumed at any time if fully controlled ventilation is re-established.

During ECCO_2_-R, patients will be initially managed with volume-control ventilation: tidal volume will be 3-5 ml/kg PBW, respiratory rate will be titrated to ensure adequate CO_2_ washout.

In the control group, PEEP will be set in order to achieve a plateau pressure of 23-25 cmH_2_O; in the intervention group, PEEP will be set according to the strategy described above for the intervention group.

The assigned treatment will be carried on while the patient is managed with fully controlled ventilation (defined as the absence of inspiratory swings in the airway/esophageal pressure tracing during a 15-second end-expiratory occlusion): during spontaneous breathing, PEEP will be set according to the decision of the attending physician.

However, in order to standardize the treatments, moderate PEEP (10-15 cmH2O) will be encouraged in case of moderate hypoxemia (PaO_2_/FiO_2_<200 mmHg) in control group, while PEEP close to the value set during controlled ventilation according to the treatment protocol but <15 cmH2O will be suggested in the intervention group.

During assist/control and assist ventilation, PEEP will never be higher than the last PEEP set according to the assigned protocol during controlled ventilation.

The choice about the timing of spontaneous breathing initiation and weaning from ECCO_2_-R will be left to the attending physician.

The PEEP-setting protocol according to the assigned treatment will be re-established at any time if fully controlled ventilation is resumed.

The use of inhaled nitric oxide will not be allowed in any of the two study groups.

***Duration of assigned treatment***

In both groups, the assigned ventilation protocol will be followed for a minimum of 72 hours from randomization and any time fully controlled ventilation is deemed necessary by the attending physician up to 14 days from randomization. After 14 days from randomization, PEEP will be set according to the clinical practice of each institution.

After 72 hours from the study protocol, the PEEP setting protocol according to the assigned treatment will be resumed at any time within 14 days from enrolment if fully controlled ventilation is established, according to the decision of the attending physician in charge.

In particular, controlled ventilation with no or minimal inspiratory effort will be encouraged in intubated patients at any time during the ICU stay if:

PaO_2_/FiO_2_ is lower than 120 mmHg with PEEP≥5 cmH2O.

Patient’s respiratory rate is higher than 35 breaths per minute while receiving mechanical ventilation and adequate sedation.

Tidal volume during spontaneous breathing exceeds 8 ml/kg PBW.

Fully controlled ventilation will be mandatory in intubated patients at any time during the ICU stay if:

PaO_2_/FiO_2_<100 mmHg.

FiO_2_≥0.7 is needed to achieve the oxygenation target.

***Spontaneous breathing***

Patients’ management with assist/control and assist ventilation will be allowed after 72 hours from the enrolment if deemed appropriate by the attending physician.

During spontaneous breathing, assist/control ventilation or assist ventilation, PEEP will be set according to the decision of the attending physician and the practice of each institution: however, in order to standardize the treatments, moderate PEEP (10-15 cmH_2_O) will be encouraged in case of moderate hypoxemia (PaO_2_/FiO_2_<150 mmHg) in control group, while PEEP close to the value set during controlled ventilation according to the treatment protocol but <15 cmH_2_O will be suggested in the intervention group.

During assist/control and assist control ventilation, PEEP will never be higher than the last PEEP set according to the assigned protocol during controlled ventilation.

Fully controlled mechanical ventilation will be resumed any time during the study period if the patient meets the criteria described above or any time deemed necessary by the physicians in charge.

***Weaning***

In order not to delay weaning from mechanical ventilation, when a patient is managed with assist/control or assist ventilation with PEEP higher than 8 cmH_2_O, a daily PEEP weaning trial will be performed whether PaO2/FIO2 ratio>150 mm Hg and FIO2<0.5: PEEP will be decreased to 8-5 cm H2O and arterial blood gas will be sampled after 20 -0 minutes. Previous ventilatory settings will be resumed if during the procedure transcutaneous oxyhemoglobin saturation decreases below 88%, PaO2/FIO2 falls below 150 mm Hg or if the patient experiences abnormal changes in respiratory rate or other clinical signs suggestive of respiratory distress.

When PaO2/FIO2 is no lower than 200 mm Hg at PEEP≤8 cmH_2_O, the patient will be considered to have acceptable gas exchange on 8-5 cmH2O of PEEP and will be deemed capable to tolerate this setting.

A 30-120-minute spontaneous breathing trial will be initiated as the following criteria are met and whether a patient tolerated fully assist ventilation with PEEP≤8 cmH2O for at least 4 hours without experiencing hypoxemia (SpO2<88% ora PaO2/FiO2<150mmHg):

improvement or resolution of the underlying cause of acute respiratory failure

normal sensorium (alertness and ability to communicate),

correction of arterial hypoxemia (PaO_2_ ≥ 60 mmHg at a FiO_2_ ≤ 0.4 with PEEP ≤ 8 cmH2O);

absence of fever (≥ 38 °C) or sepsis;

blood hemoglobin concentration of 7 g/dL or more;

hemodynamic stability without cardiac ischemia or arrhythmias (norepinephrine<0.1 gamma/kg/min).

For the purpose of the study, success of the spontaneous breathing trial will be defined as presence of the following criteria:

respiratory rate < 35/min,

arterial oxygen saturation ≥ 90%,

heart rate < 120/min,

systolic blood pressure > 90 and < 160 mmHg

adequate cough.

If the spontaneous breathing trial is successful, the patient will be extubated.

In case of SBT failure, mechanical ventilation will be resumed (with any PEEP levels accepted, according to the strategies described above) and new PEEP-weaning and spontaneous breathing trials will be performed on a daily basis.

The use of pressure support ventilation and proportional assist ventilation, as compared to assist-control modes, will be encouraged during the weaning phase.

***Extubation***

Each extubated patient will undergo oxygen therapy via high flow nasal cannula (maximum flows tolerated and FiO_2_ titrated to obtain 96%>SpO_2_>92%). Pre-emptive noninvasive ventilation (NIV) after extubation will be allowed in prolonged to wean patients (i.e. more than 3 SBT failure or more than 7 days from the first SBT to being extubated) if deemed necessary by the physician in charge[25–27].

In case of respiratory failure during oxygen therapy via high flow nasal cannula after extubation and, a rescue NIV trial will be allowed before intubation in both groups at the discretion of the attending physician.

***Tracheostomy***

Tracheostomy to facilitate the weaning process will be performed after 14-21 cumulative days of invasive mechanical ventilation.

***General note***

The daily cumulative doses of vasopressors (norepinephrine, adrenaline, dobutamine), sedative and analgesic agents (sufentanil, propofol, midazolam), the total amount of administered crystalloids and colloids, the use of diuretics, diuresis and net fluid balances will be calculated for each patient and recorded.
