# Supplementary material for: Individualized positive end-expiratory pressure guided by end-expiratory lung volume in early acute respiratory distress syndrome: study protocol for the multicenter, randomized IPERPEEP trial
Source: Trials. 2022 Jan 20;23:63. doi: 10.1186/s13063-021-05993-0 (PMC8772175; doi:10.1186/s13063-021-05993-0)
Supplement: Supplementary file 4 — Additional file 4: Scoring system for the primary endpoint. [file 13063_2021_5993_MOESM4_ESM.docx]

**Additional file 4:** Scoring system for the primary endpoint

|  | ICU Mortality | | VFD60 | IL6AUC | Score | |
| --- | --- | --- | --- | --- | --- | --- |
| Scenario | IPERPEEP | Control | Iperpeep vs. Control | Iperpeep vs.  Control | Iperpeep | Control |
| 1 | Yes | No | >,=,< | >,=,< | -1 | 5 |
| 2 | No | Yes | >,=,< | >,=,< | 5 | -1 |
| 3 | No | No | > | >,=,< | 3 | 0 |
| 4 | No | No | < | >,=,< | 0 | 3 |
| 5 | No | No | = | > | 0 | 2 |
| 6 | No | No | = | < | 2 | 0 |
| 7 | No | No | = | = | 1 | 1 |
| VFD60 will be considered > or < if the difference is > 5 days; difference<5 days will be considered = for the purpose of the scoring.  IL6AUC will be considered > or < if the difference is > 10% of the lower of the two compared values; difference<10% of the lower of the two compared values will be considered = for the purpose of the scoring. | | | | | | |
