# Supplementary material for: Individualized positive end-expiratory pressure guided by end-expiratory lung volume in early acute respiratory distress syndrome: study protocol for the multicenter, randomized IPERPEEP trial
Source: Trials. 2022 Jan 20;23:63. doi: 10.1186/s13063-021-05993-0 (PMC8772175; doi:10.1186/s13063-021-05993-0)
Supplement: Supplementary file 5 — Additional file 5: Sample size determination: assumptions and methodology. [file 13063_2021_5993_MOESM5_ESM.docx]

**Additional file 5:** Sample size determination: assumptions and methodology

The assumptions and methodology used to arrive at the determination of the sample size for the IPERPEEP study are detailed below. Henceforth, we will designate as 'Control' the conventional treatment scheme and as 'Active' the IPERPEEP new treatment.

**Assumptions**

The endpoint chosen for the study is a composite endpoint constructed as follows: Mortality in ICU (ICUdeath), 60-day ventilation-free days (VFD60) and the 72h area under the curve of the representative citokine IL-6 (IL6AUC) are the component endpoints, which are observed for each patient, in this order of importance. Each patient in the Active group is compared with every patient in the Control group and both are assigned a comparison score as described in the above 'Endpoints' section of the protocol. Comparison scores are then added for each subject to obtain the cumulative score composite endpoint, which will be compared between treatment by a non-parametric two-sample test.

One of the problems in assessing sample size for composite endpoints is assessing the correlation among component endpoints. In fact, if the component endpoints are totally correlated, they manifestly characterize the same biomedical phenomenon, but there is no gain in information with respect to taking any single one of them alone. Conversely, if the component endpoints are totally uncorrelated, the gain in information is maximal, even though some medical justification would be needed in explaining what unitary phenomenon is characterized by a combination of unrelated descriptors.

In the present instance, high ICUdeath, low VFD60 and high IL6AUC are clearly related to each other, in that all are expression of the underlying respiratory dysfunction, and they are clearly not perfectly determined from one another. The problem is that the degree of correlation among the three component endpoints is not known in general. We follow therefore an approximate, stepwise approach.

It has been taken (consistently with data reported in [1]) a ICU mortality rate of 35% in the Control group, and assumed a mortality rate of 27% in the Active group, given that a 20-25% relative reduction in ICU-mortality by PEEP setting appears reasonable[2].

By considering any non-survivor worse than any survivor, and by considering non-survivor outcomes equally unfavorable, no further consideration is given to the distribution of VFD60 and IL6AUC in non-survivors.

There is literature [3] to support the assumption that VFD60 may equal approximately 34 ± 16 days in Control and 28 ± 16 days in Active subjects. Therefore Gaussians and standard deviations can be used with these means to approximate the distribution of VFD60 given ICU survival.

Ideally, it is aimed to have observations from which to estimate the joint distribution of VFD60 and IL6AUC in survivors under Control and Active treatments in order to be able to extract from it the conditional distribution of IL6AUC given any subject's VFD60. Since this is not possible, it was observed that (as can be expected), VFD60 and IL6AUC are inversely related when considering their average values in two groups of subjects( [3] Bein et al. Control and Active):

estimated IL6AUC = 14753 - 264 * VFD60 hrs * pm / ml

(setting negative values to zero).

It was possible to exploit this inverse relationship in order to assume a determined value of IL6AUC at any given value of VFD60 (in which case these two component endpoints would be assumed to be totally correlated), or to assume a value for IL6AUC distributed randomly, independently of VFD60 (in which case the two component endpoints would be assumed to be totally uncorrelated).

Instead, it is assumed that IL6AUC to be normally distributed around its conditionally expected value given VFD60 (obtained through the relationship above) with a standard deviation of 5750 hrs * pm / ml, consistent with the interquartile ranges reported by Bein et al [3].

**Procedure**

Under the above assumptions, for each sample size 2N, M virtual trials have been simulated. For each virtual trial, the component endpoints for N virtual patients in each arm have been randomly generated according to the above assumptions, the composite endpoint has been computed for each virtual patient and the two samples of composite endpoints have been compared with a Wilcoxon-Mann-Whitney rank-sum test. The proportion of virtual trials yielding a statistically signicant Wilcoxon at the α = 0.05 Type 1 error level tends to the actual power of the projected clinical trial as M tends to infinity.

**Results**

The following tables report the empirically computed powers using respectively 2000, 5000 and 10000 virtual trials for each N. Each Table-figure shows the results of three different power computations:

the power achievable using ICU mortality as endpoint (with an exact Fisher test between the two groups);

the power achievable using a simple score equal to survival*100 + VFD60 (with a WilcoxonMann-Whitney test between the two groups);

the power achievable using the composite endpoint defined above (with a Wilcoxon-MannWhitney test between the two groups).


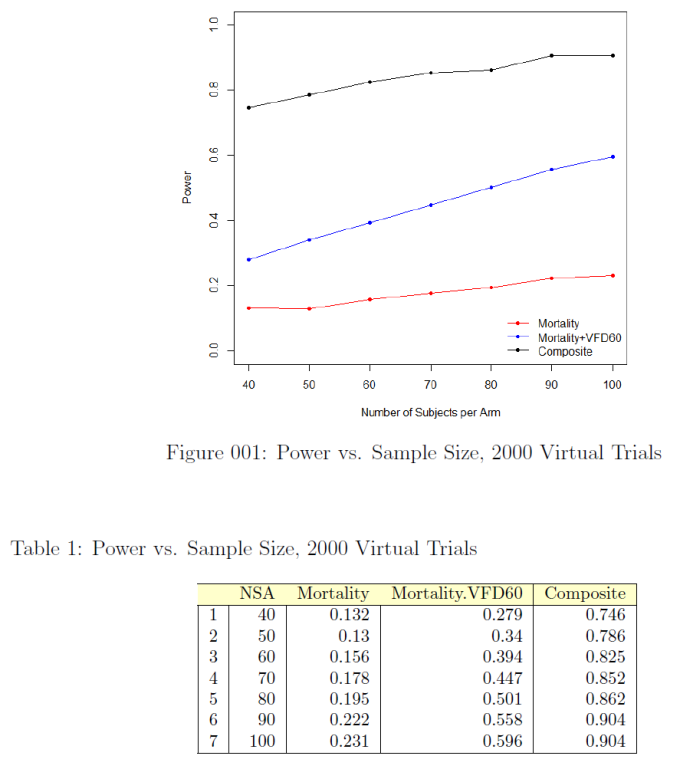


Table 1- Figure 1. Power computations over 2000 simulated trials. NSA indicates Number of Subjects per Arm.


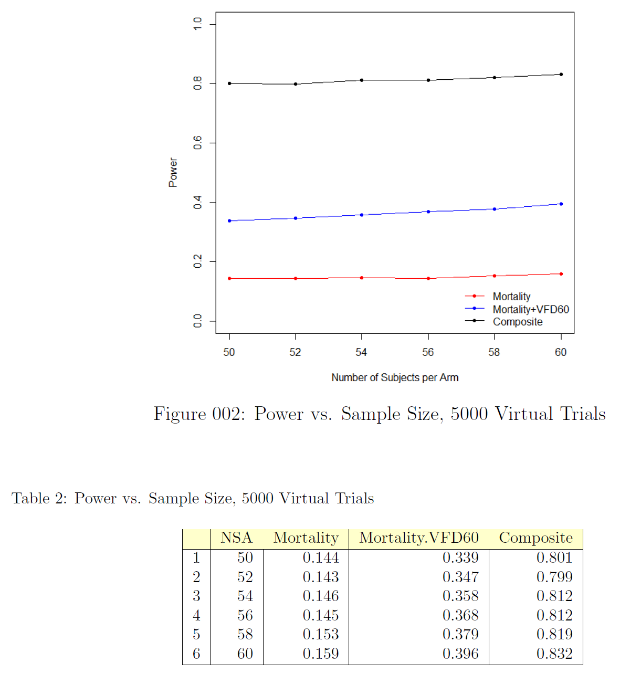


Table 2-Figure 2: power computations over 5000 simulated trials


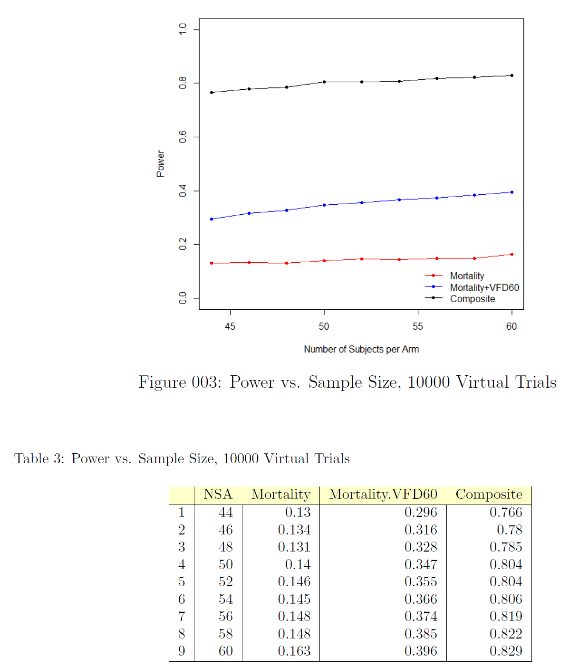


Table 3: power computations over 10000 simulated trials.

**Conclusions**

Under the stated assumptions, given the results obtained, and adopting a conservative approach in order to compensate for the small residual random variation in the numerical assessment of the sample size, it appears that a sample size of 56 subjects per arm would be sufficient to obtain 80% power in detecting a difference between Control and Active if the composite endpoint is used, at a Type 1 error level of 0.05, two-tail. With an attrition rate of 15%, a total of 132 patients in two equal groups of 66 patients each should be enrolled.

References

1. Bellani G, Laffey JG, Pham T, Fan E, Brochard L, Esteban A, et al. Epidemiology, Patterns of Care, and Mortality for Patients With Acute Respiratory Distress Syndrome in Intensive Care Units in 50 Countries. JAMA [Internet]. 2016;315:788–800. Available from: http://www.ncbi.nlm.nih.gov/pubmed/26903337

2. Briel M, Meade M, Mercat A, Brower RG, Talmor D, Walter SD, et al. Higher vs lower positive end-expiratory pressure in patients with acute lung injury and acute respiratory distress syndrome: systematic review and meta-analysis. JAMA [Internet]. 2010 [cited 2014 Jul 24];303:865–73. Available from: http://www.ncbi.nlm.nih.gov/pubmed/20197533

3. Bein T, Weber-Carstens S, Goldmann A, Müller T, Staudinger T, Brederlau J, et al. Lower tidal volume strategy (≈3 ml/kg) combined with extracorporeal CO2 removal versus “conventional” protective ventilation (6 ml/kg) in severe ARDS: The prospective randomized Xtravent-study. Intensive Care Med. 2013;39:847–56.
